# Effect of Heamolysis on Prostate-Specific Antigen

**DOI:** 10.5402/2012/729821

**Published:** 2012-12-01

**Authors:** Hasan S. Sağlam, Osman Köse, Fatma Özdemir, Öztuğ Adsan

**Affiliations:** ^1^Department of Urology, Sakarya University, Medical Faculty, Sakarya, Turkey; ^2^Department of Urology, Sakarya Training and Research Hospital, Kemalpaşa Mah, 222 Sokak, ATA-8, Sitesi, No. 9/3, Serdivan, Turkey; ^3^Department of Biochemistry, Sakarya Training and Research Hospital, Sakarya, Turkey

## Abstract

*Purpose*. We have investigated the effect of haemolysis on free and total prostate-specific antigen (PSA) in daily clinical practice. *Materials and Methods*. Thirty-nine consecutive men were enrolled in this study. With an 18 gauge (G) needle 4 cc of blood samples were drawn from the right arm and 2 cc of it was expelled gently in a Vacutainer for regular PSA assay and the remaining was emptied into a second tube for complete haemolysis. Simultaneously 2 cc of more blood were taken with a 26 G insulin needle from the left arm of the same patient and expelled into another Vacutainer with forcing. All three samples were assayed for free PSA (fPSA), total PSA (tPSA), and potassium (K). *Results*. The results of the first tube were fPSA 0,535 ng/mL; tPSA 2,493 ng/mL; K^+^ 4,178 mmol/L. The results from the haemolysis tube were 0,170 ng/mL; 0,929 ng/mL; 39,545 mmol/L for fPSA, tPSA, K^+^, respectively, (*P* value was 0,001 for all the changes). In the same order the third tube results were 0,518 ng/mL, 2,322 ng/mL, and 7,11 mmol/L. *Conclusions*. Haemolysis may result in interference by decreasing free and total PSA falsely in daily blood draw practice, that could lead to misinterpreting the case in which especially small amount of increase may be of value.

## 1. Introduction

Discrimination between benign and malignant conditions of the prostate has been made mainly by using digital rectal examination and PSA elevation, and a suspected malignancy has been confirmed by biopsy of the prostate due to poor specific and sensitive properties of PSA. PSA and its derivatives, although having some limitations, have been extensively used in prostate cancer detection, staging, and followup of the patients after treatment [1, 2]. 

While it has also been a subject of an extensive debate in diagnosis and screening the prostate cancer, followup of the patients was achieved with less debate by use of PSA and its derivatives [3–5]. Management of cases may be apparent provided that PSA levels are markedly over or below the cutoff value, but the state that PSA values are around the cutoff value has been a challenge for urologists [6, 7]. In such conditions little changes of PSA or its derivatives, produced by hidden factors related to either biological variation of PSA or laboratory analysis, may have inevitable effect on selecting patients for prostate biopsy [8, 9]. 

Herein, we present a study about probable effects of blood draw method on free and total PSA to investigate if blood collection method is of value in clinical practice.

## 2. Materials and Methods

After local ethics committee approval, male participants between 40 and 84 years of age (mean 61 ± 12) were enrolled in this study. Patients with any liver and blood disease that could cause haemolysis, hyperlipidemia, active infection of any system, antibiotic therapy, indwelling catheter, a history of urological instrumentation, or operation carried out recently were excluded from the study. After elimination of the patients according to the inclusion criteria, the remaining eligible 39 patients were included in the study. Blood draw was performed in room temperature conditions. In the morning 4 cc of blood were drawn gently from the right arm with a straight 18 G needle and the blood was emptied slowly into two Vacutainers equally. Meanwhile a tourniquet was applied to the right arm and as soon as the vein entered, the tourniquet left to allow the pooled blood flow away for 5 seconds and later the sample was taken. With a 26 G insulin needle 2 cc of blood also was taken from the left arm of the same patient and emptied with pressure into a third Vacutainer. For the left arm the tourniquet stayed till blood draw ended. The latter sample was considered as a model for a possible daily practice that could be a cause for haemolysis. 

The three tubes, each containing 2 cc of blood samples taken at the same time from the same patient, were delivered to the laboratory without delay. The first tube containing the blood taken with 18 G needle was accepted as the control that an optimal model for blood draw. The sample in the second tube, comprising the second half of the blood of the first tube, was heamolysed completely to have an almost impossible but an ultimate example of haemolysis in clinical setting and assayed for PSA and K measures. The third tube containing the blood taken with 26 G needle was used as a model of haemolysis that could be a possible example of error in clinical setting. The laboratory staff were blind for the first and the third groups. All samples were assayed for fPSA, tPSA, and potassium (K). Free and total PSA were measured only by chemiluminescent-based method (Kemiflex, Abbott Diagnostics, Illinois, USA). Potassium was used to exhibit the haemolysis of the blood. Among these patients, seven men having PSA values above 4.000 ng/mL have been advised prostate biopsy but their consequences were kept beyond this study.

The measures of all the groups were distributed normally but we intended to compare the repeated measures of the same patients to exhibit effects of haemolysis. Therefore “paired *t*-test” was applied. The results were evaluated using the Statistical Package for the Social Sciences (SPSS) version 20.0 (SPSS Inc., Chicago, IL, USA). A *P* value <0.5 was considered statistically significant. 

## 3. Results 

According to the results summarized in Table 1, tPSA values obtained from the blood drawn with 26 G needle decreased to 2,322 ± 0,588 ng/mL (7% decrease) when compared to normal serum values (*P* = 0.001). f/t PSA ratio changed from 21,463% to 22,31% because changes of the analytes were not parallel to each other. Similarly fPSA and tPSA values decreased to 0,170 ± 0,043 ng/mL (68% decrease) and 0,929 ± 0,299 ng/mL (63% decrease), respectively, from the blood haemolysed completely (*P* < 0.001). Free to total PSA ratio changed into 18%. Haemolysis causing alterations was demonstrated by obvious increment of potassium as summarized in Table 1 (*P* < 0.001 for both 26 G needle induced and complete haemolysis). 

## 4. Discussion

Some effort has been exerted by using fPSA, cPSA, PSA velocity, PSA density, urinary PSA, and so forth, to prevent unnecessary biopsies [3, 10, 11]. Factors affecting PSA levels, other than prostate cancer such as analytic and biologic and as well as relating to nonmalignant lesions like prostatitis or some urologic manipulations may even result in a confusing state for urologists in particularly borderline cases [8, 12–14]. 

Haemolysis, the breakdown of erythrocytes, has been defined as the major cause of interference altering the correct value of an analyte of interest, resulting in misinterpreting the clinical condition. It mainly occurs in vitro conditions, at the time of sample collection or transport [15]. Lippi et al. stated that small bore needles for collecting blood and shaking samples, with some other factors, compromised blood cells integrity causing intracellular contents leakage into the media and producing biological and analytical interference [16]. As stated we have shown both free and total PSA were affected by a mild haemolysis caused by 26 G needle but as shown in Table 1, change of tPSA was not exactly parallel to that of fPSA. Haemolysis was confirmed by exhibiting a 1.7-fold increase in K^+^ concentration. Mild haemolysis was described as a lysis of hemoglobin up to 0.2 g/dL of concentration resulting a nonvisible hue in the serum [17]. As opposed to the literature, our results show an interference by haemolysis has taken place as a slight decrease in the results of measuring analytes fPSA and tPSA [17, 18]. This effect was strongly visible with the complete haemolysis as results of measuring fPSA and tPSA decreased by 68% and 63%, respectively. On the contrary Snyder et al. stated that by using chemiluminescent-based method, as we did, they had a large increase in results of Troponin-I and PSA even in moderate haemolysis [17]. Therefore their results are inconsistent with those we obtained. To see the direction of effect better we did maximize the haemolysis as hemolysing the sample completely and saw the direction persisted consistent as a decrease of PSA, free and total. As stated previously, we have seen that gross haemolysis caused more effect than slight haemolysis. Thus the low levels of analyte concentration exhibited low false change but this change may be of great importance in some instances such as borderline cases for biopsy or prostate cancer followup after treatment.

Although exact mechanisms of interference with immunoassays are not known yet, many factors proposed to cause interference such as analyzing methods like immunoflorescence or photometric methods, haemolysis, paraproteinemia, hyperlipidemia, hyperbilirubinemia [18]. Type of effect as negative or positive also is dependent on the type of analyte of interest and as well as being intracellular like potassium or extracellular. For haemolysis, hemoglobin itself can cause an interference even in chemiluminescence that is stated as highly sensitive and specific tracer so the less prone to erythrocyte lysis [15]. Additionally when erythrocytes lysed, the contents including structural proteins, enzymes, lipids, carbohydrates, and other materials might interact or compete with assay reagents [16]. Interference may also be due to less specific reagent antibodies those cross-reacting with some other contents released from the damaged cells or some compounds released from the cells may bind to the analyte and inhibit antibody-binding sites [17]. On the other hand Lindstedt et al. reported that PSA did not change due to visually detected haemolysis with immunoradiometric method (with Tandem-R, PSA kit) [18].

Serum PSA concentration was stated to be one important factor while at lowest levels, for the greatest effect. Therefore the higher serum PSA levels seemed affected less from haemolysis [17]. The results we have obtained revealing similar effect for both lower and higher concentrations of analytes are not consistent with that observation. 

Because fPSA did not change in parallel with tPSA, the initial ratio of f/t PSA increased slightly from 21,46% to 22,30% but the ultimate value decreased to 18,30%. As tPSA decreasing falsely, an increase of f/tPSA in mildly haemolysed blood that is likely in clinical practice may be an additional cause of misinterpreting for borderline PSA values or posttreatment followup. Of course most laboratories comment on haemolysis and the test may be repeated but it should be kept in mind that haemolysis is not a dichotomous event, therefore some degree of haemolysis, even if assumed acceptable, may be examined. 

### 4.1. Limitations 

However, the present study is based on a consecutive 39 patients who have been referred to our hospital on the same day with some complaints, urological or not. A power analysis and number of participants could not be calculated. This method is used to collect and handle the blood with exactly the same two good-audit nurses to keep the handling strictly as planned because the study is about the preanalytical handling the samples. Nevertheless it cannot be excluded that this selection type has included some bias.

## 5. Conclusion 

In vitro preanalytical haemolysis may result in significant interference even in chemiluminescence methods. Needle bore and its structure and as well as handling the blood after draw may be effective factors in daily blood collection practice, reducing PSA levels and increasing f/tPSA falsely. To quantify the effects in respect to needle properties and following handle the samples, more investigations may be required. 

## Figures and Tables

**Table 1:** Complete results of each group.

	Samples, by		*P*	Complete haemolysis
	18 G	26 G		
fPSA (ng/mL) Mean ± SD	0.535 ± 0.191	0.518 ± 0.189	0.001	0.170 ± 0.043
tPSA (ng/mL) Mean ± SD	2.493 ± 0.562	2.322 ± 0.578	0.001	0.929 ± 0.299
f/tPSA (%)	21.46	22.31	0.001	18.30
K^+^ (mmol/L) Mean ± SD	4.178 ± 0.511	7.11 ± 2.576	0.001	39.545 ± 5.775
